# BUILDING INDIGENOUS HEALTH: INSIGHTS FROM INDIGENOUS ADULTS ON KNOWLEDGE INTEGRATION AND ADAPTIVE STRATEGIES

**DOI:** 10.1093/geroni/igae098.1826

**Published:** 2024-12-31

**Authors:** Zih-Yong Liao, Fang-Lin Kuo

**Affiliations:** National Yang Ming Chiao Tung University, Taipei, Taiwan (Republic of China); National Health Research Institutes, Yunlin, Yunlin, Taiwan (Republic of China)

## Abstract

Over half of the older population faces inadequate dietary intake, posing higher risks of geriatric syndromes like frailty and sarcopenia. This community-based participatory research (CBPR) co-created a nutrition and cooking workshop in a Taiwanese Indigenous community. Employing the photovoice method alongside focus groups and individual interviews with 16 participants, we sought to understand the older Indigenous adults’ dietary patterns. Nutbeam’s health literacy model (2000) and Giddens’ structuration theory (1984) guided data analysis and conceptualization of food literacy construction in the Indigenous land. Four main themes emerged. First, ‘knowledge integration’ represents the conversation between traditional and scientific food/nutrition knowledge, strengthening functional food literacy to address health concerns. Second, ‘Indigenous adaptive strategies’ demonstrate their ability to apply food knowledge meaningfully in daily life, representing interactive food literacy. Third, ‘awareness of advanced nutrition literacy’ demonstrates the older adults’ ability to evaluate personal diets and critically reflect on the need for better health, representing critical food literacy. These food literacy constructs are iterative and interactive. Lastly, work is central to Indigenous life, shaping their identity and a sense of achievement, motivating older adults’ adaptive strategies and pursuing advanced nutrition literacy. However, the middle-aged population is less likely to attend health promotion activities due to work. Indigenous adaptive strategies reflect human agency’s potential to reproduce individual food ecosystems and healthcare practice systems within the community, overcoming structural barriers. CBPR can foster a sense of ownership among participants, enabling them to engage healthy behaviors as their personal priorities and take active initiatives toward better health.

